# Supplementary material for: Helicobacter pylori resistance to antibiotics before and after treatment: Incidence of eradication failure
Source: PLoS One. 2022 Apr 20;17(4):e0265322. doi: 10.1371/journal.pone.0265322 (PMC9020706; doi:10.1371/journal.pone.0265322)
Supplement: S1 Protocol — (DOCX) [file pone.0265322.s001.docx]

# Studyprotocol

# Chronic HP infection, immunodeviation and malignancy potential

## Responsible for the project

Cand. med., Consultant Oddmund Nestegard, Medical Department Callingike Hospital, Vestre Viken /Laboratorium for Gastroenterology and Nutrition.

This project will be applied for as a PhD project at the University of Tromsø.

## Employees/supervisors:

Prof. dr. med., head of section Jon Florholmen, Gastromed. avd, UNN/UiT

Associate Professor Dr. med., Consultant Physician Eyvind J.Paulssen, Gastromed. Avd., UNN

Dr. Med. Const. consultant, Gastromed. Avd., UNNRasmus Goll

## Staff:

## Dr. med., ass. physician Anne Mette Asfeldt, Medical Dec., UNN

## Dr. med., Senior Researcher , UiTCui Guanglin

Dr. med., Consultant Physician Sonja Eriksen, Department of Pathology, UNN

Dr. med. Tore Lier, Microbiological Avd., UNN

## Hypotheses

Prolonged infection with helicobacter pylori (HP) may result in altered immunology that may increase

risk ofmalignancy. This may be due to properties of the bacterium and/or in the host.

***Main goal***

Describe immunological changes in gastric mucosa in a long-term HP infection and their relationship to antibiotic resistance to the bacterium and cancer development

***Sub-objective 1:***

Clinical phenotyping of people with antibiotic resistance to HP and microbiological phenotyping of HP strains

***Milestone 2***

Immunological characteristics in gastric mucosa in patients with prolonged HP infection and antibiotic resistance and association with immunological markers associated with cancer development in gastric mukosa.

## Background

- 1. *Helicobacter pylori infections clinic*

The bacterium Helicobacter pylori (HP) was detected in 1983 and has been the main cause of the gastric ulcer disease or rather peptic ulcer, for several hundred years. (Marshall BJ et al. Lancet 1984, 16, 1311). This discovery revolutionized understandingone of the peptic ulcer disease, and this gave the start of an antibiotic treatment era in which now ulcers caused by the bacterium are a rare disease in Western countries. However, HP infection globally is a major problem with prevalens up to 90% in some underdeveloped countries. In Norway on the other hand, prevalence was down to 40 % in the Sørreisa survey where 12 % were associated with ulcers (Bernersen B et al Gut 1990, 31, 989).

HP infection causes gastroduodenitis and ulcer pepticum. Another important condition associated with HP infection is ventricular cancer. It is now well documented that hp infection can give rise to stomach cancer through the sequence of chronic gastritis- atrophic gastritis, intestinal metaplasia, dysplasia and ventricular cancer (Fox and Wang, J Clin Invets 2007,)

*1.2. Helicobacter pylori and treatment*  *resistance*

HP infection is traditionally treated with antibiotics, acid-retardant drugs and bismuth in various combinations. The eraction rate varies between 70-90%. Recent studies show declining eraction rates. Something can be explained by increasing antibiotic resistance. A study has shown that the combination of antibiotic resistance and lack of compliance accounts for about 40% of the lack of efficacy of treatment. The remaining 60% have other causes. Somewhat surprising is to observe that this patient cohort is not characterized clinical phenotypic while antibiotic resistance is incompletely mapped. The mest widespread resistance is metronidazole that can be detected in vitro. However, there is little benefitfrom resistance examinations and is not routinely used today. However, a cohort of antibiotic-resistant patients has gradually been generated. They sometimes get recidiv of ulcers that are treated with acid-retardant treatment. The big question is whether these patients eventually get the development of cancer ventriculars.

- 1. *Chronic Helicobacter pylori infection and immune response*

The immune response to microbes is initially an unspecific immune response - the so-called *innate immune system*- initiated by the fact that the microbe has molecular determinants that are recognized by our immune system as "alien" by the so-called pattern recognition receptors (Toll like receptors, NODs etc). The immune response is then directed towards an antigen-dependent reaction with the development of T-cell response. The T-cell response can be directed in the direction of a TH1 direction i.e. TH-1 helps cell reaction which is typical reaction to most microbes and a TH2 response that is typisk for a few microbes but ellers typical of a more allergic reaction and autoimmunity. In the case of HP infection, a TH1 response has previously been described, while studies at Gastroenterologyand laboratory,UiT are described as a combined TH1 and TH2 immune response (Goll R et al. Helicobacter 2007, 12, 185). After an HP eradication, the mucous membrane inflammation will gradually disappear and the associated immune response will be reduced. In some add-ons,the inflammation develops in a more TH2 direction with autoimmune gastritis, which is the precursor to the premature state of atrophic gastritis. Whether all atrophic gastritis passes through an autoimmune condition has not been clarified.

- 1. *Chronic Helicobacter pylori infection, immune response and cancer development*

The mechanisms behind the development of cancer ventricules associated with HP infection have not been clarified. On the other hand, it is assumed that the immune system plays a significant role in both the resistance to cancer development and carcinogenic development. It is believed that cancer ventriculars start with a mutating cell in ventricular mucosa. The types of cells that mutate have not yet been clarified. Our immune apparatus will then react against this foreign cell with a TH1 response. The hypothesis that the Gastroenterological Laboratory, UiT researches is that if one develops cancer, one sees a TH2 deviering of the immune response either as a direct cause of or as a result of the cancer (for an overview, see Cui G and Florholmen J et al Inflammation & Allergy – DrugTarget 2008, 2008, 7:94). Last year's research has yielded new immunological phenotypes associated with HP associated with cancer ventriculars. Shuiping T and co-author have shown in a transgenic mouse model that HP induces ventricular cancer via an interleukin (Il)-1 beta mechanism (Cancer Cell 2008, 14, 408). Various clinical studies have suggested polymorphism in various pro-inflammatory cytokines such as Il-1beta, TNF alpha, and Il-6 (for an overview, see Shuiping T). The newly discovered Il-33 (Th2 family) cytokine is normally found in vascular epithelial cells but disappears by cancer development (Küchler AM et al 2008, Am J Pathol 173, 1229). Furthermore, TNFR1/Il-17/Il-23 is associated with ovarian cancer development in an animal model (Charles KE et al. J Clin Invest 2009, 119, 3011) and polymorphism of the Il-17 gene are associated with gastric carcinogenese (Shibata T et al. Hum Immunol 2009, 70, 547).

*1.5. Background and objective of the study.*

The interaction between bacterium and host in chronic HP infection is complex, Possibly some immunological mechanisms can explain immunological phenotypes such as antibiotic resistance and/or cancer development. With this study, we will try to shed light on whether there are immunological factors in the gastric mucosa that may increase malignancy potential.

One will also try to shed light on immunological conditions in the host's gastric mucosa and conditions of the bacterium that may provide explanations for the lack of effect of eraction treatment.

One will look at the resistance conditions of the bacterium and see if there has been a similar development of resistance in the Norway.

## MATERIAL AND METHODS

**Material and pre-axe design- see otherwise Floatscheme**

Patientmaterial contents of one main group and five control groups.

1. The main material: 40 patients from UNN who have been treated for upper GI tract disease caused by HP infection found by gastroscopy and detected by urease rapid test in the time 1/1-96 -31/12-2002. These patients have received treatment for the HP infection. Those who are still HP-positve after 2 treatments with antibiotics are defined as HP-treatmentresistant. These 40 patients receive a request to participate in the study - a number of 30 patients are expected to participate.

- Characterize clinical phenotype: Ulcus ventriculi, ulcer duodenia, gastritis, sex, age
- Gastroscopy with biopsytaking for analysis histologically, microbiologically and immunologically
- Offer of new treatment after resistance determination
- Control gastroscopy with biopsies after 3 months. for histologicale, bacteriological and immunologicale analyses

1. Control groups on every 30 patients (applies to both Vestre Viken HF Ringerike Hospital and UNN except group D). :

- A) Previous (> 5 years) HP erad. These are recruited from references to gastroscopy where it emerges that they have previously been diagnosed with the HP infection. These are offered gastroscopy with biopsytaking for histological, bacteriological and immunological analysis. Clinical phenotyping.
- B) Newly diagnosed HP-positive ulcus ventriculi, ulcer duodenia and erosive gastritis. Gastroscopy with biopsytaking for histological, bacteriological and immunological analysis. Offered treatment for HP infection according to routines. Controllgastroscopy 3 months. Later where biopsytaking is repeated
- C) HP negative without mukosal changes. Gastroscopy with biopsy for histological, bacteriological and immunological analysis.
- D) Persons who do not have gastrointestinal disease. Analysis of previous biopsies taken at the Sørreisa survey. Here you will use the full available material.
- E) Patients with ca.ventriculi and who are HP positive. Gastroscopy for histological, bacteriological and immunological analysis. Here the number will necessarily be fewer due to difficult recruitment of the combination HP positive and ca ventrikuli. We therefore plan 15 patients.

1. Bacteriological e-analysis r:

- Detect the bacterium
- Extended resistance determination (amoxicillin, claritromycin, mitronidazole, fluoroquinolones, rifampins)

1. Histological analysis (Pathological department UNN/ pathological department, Buskerud Hospital

- Proliferationindex – Ki67
- Sydneyklassification
- Immunological e-analysis:
- Immunhistokjemi
- PCR analysis of cytokines
- Protein analysis of cytokines
- Single cell analysis (on selected UNN patients).

At the Laboratory of Gastroenterology and Nutrition, the following methods are available for the examination of biopsies: :

"Real time" quantitative measurement of mRNA (RT-PCR) (in liver biopsies); TNF-alpha, IL1-, IFN-gamma, IL-4, IL-6, Il-8, IL-12, Il-17, Il-23, Il-33, transcription factors TBX21, GATA3, and ROR-gamma and BCL-2, Bax. HP associated Cag A and Vac A. Immunohistochemical techniques for the detection of various cytokines, apoptosis and various immune-active cells (CD4, dendrite cells, macrophages, activated fibroblasts). Magnetic assisted cell sorting (MACS) for functional analysis of cells isolated from ventricular biopsies

*statistics.* Similar statistical methods are used as in the clinical and molecular biologist published articles, see CV Jon Florholmen

**statistics**

Different statistical methods will be used to assess clinical and laboratory data - this is stated in previous publications for dr with Rasmus Goll and dr with Anne Mette Asfeldt - see the publication list of Jon Florholmen. It revolves around student T test, Wilcoxon sum rank test, Kruskal-Walls test, Mann-Whitney test with Bonferroni corrections and Spearman's correlation test.

**Approvals**

Approval is sought from the Regional Committee (Nord Norway) while biological data bank has already been established (HP study to dr with Rasmus Goll).

## Status october 2009

Collected data for 7 years of material in the period 1/1-96 – 31/12-02 obtained from the journal system at UNN. There are patients who have either been admitted or been to outpatient treatment and who have been diagnosed with K25 – K29 in the diagnostic system ICD10 or 531.0 – 533.1 in ICD9 and who in the vast majority of cases have had gastroscopy performed (except for someone who has been diagnosed with a breath test). 4 0 patients have been registered.

One has registered gender, age of patient, type of cure where it has been possible to find, possibly several cures and whether the patient is eradicated.

3. PROJECTS

The following subprojects will be completed and evaluated:

*3.1 Clinical and microbiological phenotypes of HP treatment-resistant patients.*

It will call in the 40 HP treatment-resistant patients who have had the disease for 12-15 years after failing HP's eradication. One will characterize age, sex, ulcer disease earlier (before initial treatment) and now, annual need for the number of acid-reducing cures, *quality of life* registrations as well as the effect of new erosion treatment.

The HP bacterium will be characterized as antibiotic resistance pattern as well asdetected g of toxins (Cag A and Vac A) will be determined.

This is an original study as thecliqueand microbiological phenotypes have not been mapped for treatment resistanceofHP positive patients.

Planlagt artikkel: *Nestegard O et al.*  *Clinical and microbiological characterizations of Helicobacter pylori P infected patients 15 years after unsuccessful eradication*

*3.2. Immunological characterization of ventricular mukosa after prolonged HP infection in treatment-resistant patients.*

The hypothesis is that through a prolonged HP infection as our group of HP treatment-resistant patients represents, the immune response in ventricular mukosa will change into a premature immunophenotypic pattern.

The choice of control groups (group B-E, see above) makes such an assessment possible. The "normal" control will be HP negative patients without endoscopic changes in the upper GI funnel HP erad patients > 5 years after the erosion will be an important comparable control. The control group recently detected disease with subsequent eradication we will select patients < 50 years of age to omit those with suspected prolonged HP infection. This is not an idell control as we do not know how long they have hadthe disease. These patients will be HP eradicated and the mukosa immunology before and after treatment will give us useful knowledge in the overall assessment of the mukosa immunology of a long-term HP infection. HP infected ventricular mukosa in the patient with stomach cancer is also an important control group in the capacity that in this mukosa malignant disease has been established.

This material will be the recurrence of a descriptive immunological and bacteriological characterization. According to the findings, more specific analyses will be carried out to look at the mechanisms behind the development of the disease.

You will plan two main articles:

Nestegard O et al. Immunological phenotypes in gastric mucosa after longstanding HP infection.

Nestegard O et al. Helicobacter pylori resistance profiles and toxins in treatment sensitive an insensitive patients.

According to the above findings, more mechanistic analyses will be carried out in the perspective of malignant development on the basis of a long-term HP infection.

4: Studyprogress

3 years project. Data collection will take place from 1 January 2010 to 3112 2011. Manuscript processing will start as early as 2010 and is expected to end 31122012.

5 . Budget

Expenses for the project are mainly expenses for primary researcher (chief medical officer Oddmund Nestegard). He will need a 20% position in the first two years and a 100% position in the latter part of the research period. These expenses will be applied locally (Ringerike) as well as HelseNord.

The analysis expenses will be covered by the Gastrofondet UNN.

6. MEANING

This project will provide new knowledge in 2 main areas: new knowledge about the consequences of long-term chronic HP infection that is both nationally but especially globally a major clinical problem; the impact of a chronic HP infection on the development of cancer. This association is well documented. So far, it has been refused to remove the bacterium due to the fact that large parts of the population are infected in some parts of the world. One must therefore concentrate on treating special vulnerable groups. This study may provide more knowledge about whether there are special immunological phenotypes that are in a cancer development risk group.
