# Supplementary material for: Helicobacter pylori resistance to antibiotics before and after treatment: Incidence of eradication failure
Source: PLoS One. 2022 Apr 20;17(4):e0265322. doi: 10.1371/journal.pone.0265322 (PMC9020706; doi:10.1371/journal.pone.0265322)
Supplement: S2 Protocol — (DOCX) [file pone.0265322.s002.docx]

# Studyprotocol (in norwegian language)

# Kronisk HP infeksjon, immundeviasjon og malignitetspotensiale

## Prosjektansvarlig

Cand. med., overlege Oddmund Nestegard, Medisinsk avdeling Ringerike sykehus, Vestre Viken /Laboratorium for Gastroenterologi og Ernæring.

Dette prosjektet vil bli søkt som et PhD prosjekt ved Universitetet i Tromsø.

## Medarbeidere/veiledere:

Prof. dr. med., seksjonsoverlege Jon Florholmen, Gastromed. avd, UNN/UiT

Førsteamanuensis dr. med., overlege Eyvind J.Paulssen, Gastromed. avd., UNN

Dr. med., konst. overlege Rasmus Goll, Gastromed. avd., UNN

## Medarbeidere :

## Dr. med., ass. lege Anne Mette Asfeldt, Medisinsk avd., UNN

## Dr. med., seniorforsker Cui Guanglin, UiT

Dr. med., overlege Sonja Eriksen, Patologisk avdeling, UNN

Dr. med. Tore Lier, Mikrobiologisk avd., UNN

## Hypoteser

Langvarig infeksjon med helicobacter pylori (HP) kan gi endret immunologi som kan gi økt

risiko for malignitet. Dette kan skyldes egenskaper hos bakterien og/eller hos verten.

*Hovedmål*

Beskrive immunologiske endringer i gastrisk mukosa ved en langvarig HP-infeksjon og deres relasjoner til antibiotikaresistens mot bakterien og kreftutvikling

***Delmål 1:***

Klinisk fenotyping av personer med antibiotikaresistens mot HP og mikrobiologisk fenotyping av HP stammene

***Delmål 2***

Immunologiske karakteristika i gastrisk mukosa hos pasienter med langvarig HP infeksjon og antibiotikaresistens og assosiasjon til immunologiske markører som knyttes til kreftutvikling i gastrisk mukosa.

## BAKGRUNN

- 1. *Helicobacter pylori infeksjonens klinikk*

Bakterien Helicobacter pylori (HP) ble påvist i 1983 og har vært hovedårsaken til magesårssykdommen eller rettere skrevet peptisk ulcus, i flere hundre år. (Marshall BJ et al. Lancet 1984, 16, 1311). Denne oppdagelsen revolusjonerte forståelsen av peptisk ulcer sykdommen, og dette gav starten til en antibiotiks behandling æra hvor nå magesår forårsaket av bakterien er en sjelden sykdom i vestlige land. Imidlertid er HP infeksjonen globalt et stort problem med prevalens opp i 90 % i enkelte underutviklede land. I Norge derimot var prevalensen ned mot 40 % i Sørreisaundersøkelsen hvor 12 % var assosiert til magesår (Bernersen B et al Gut 1990, 31, 989).

HP infeksjonen gir gastroduodenitt og ulcus pepticum. En annen viktig tilstand som assosieres til HP infeksjon er ventrikkelcancer. Det er nå vel dokumentert at HP infeksjonen kan gi opphav til magekreft gjennom sekvensen kronisk gastritt- atrofisk gastritt, intestinal metaplasi, dysplasi og ventrikkel cancer (Fox og Wang, J Clin Invets 2007,)

*1.2. Helicobacter pylori og behandlings resistens*

HP infeksjonen blir tradisjonelt behandlet med antibiotika, syrehemmende medikamenter og bismuth i ulike kombinasjoner. Eradikasjonsraten varierer mellom 70-90 %. Nyere studier viser fallende eradikasjonsrater. Noe kan forklares med økende antibiotikaresistens. En studie har vist at kombinasjonen antibiotikaresistens og manglende compliance utgjør ca. 40% av manglende effekt av behandlingen. De resterende 60% har andre årsaker. Noe overraskende er det å observere at denne pasient cohorten ikke er karakterisert klinisk fenotypisk mens antibiotika resistensen er ufullstendig kartlagt. Den mest utbredte resistensen er metronidazole som kan påvises in vitro. Imidlertid er det liten klinisk nytte av resistensundersøkelser og benyttes ikke rutinemessig i dag. Imidlertid har man etter hvert generert en cohorte av antibiotikaresistente pasienter. De får av og til recidiv av magesår som behandles med syrehemmende behandling. Det store spørsmålet er om disse pasienter etter hvert får utvikling av cancer ventrikuli.

- 1. *Kronisk Helicobacter pylori infeksjon og immunrespons*

Immunresponsen mot mikrober er initialt en uspesifikk immunrespons- den såkalte *innate immune system*- som initieres av at mikroben har molekylære determinanter som gjenkjennes av vårt immun system som ”fremmed” ved det såkalte pattern recognition receptors (PPR’s)(Toll like receptors, NODs etc). Immunresponsen dirigeres deretter mot en antigen-avhengig reaksjon med utvikling av T-celle respons. T-celle responsen kan dirigeres i retning av en TH1-retning dvs TH-1 hjelper celle reaksjon som er typisk reaksjon mot de fleste mikrober- og en TH2 respons som er typisk for noen få mikrober men ellers typisk for en mer allergisk reaksjon og autoimmunitet. Ved HP infeksjon er det fra tidligere blitt beskrevt en TH1 respons- mens studier ved Gastroenterologisk laboratorium, UiT er beskrevet et kombinert TH1 og TH2 immun respons (Goll R et al. Helicobacter 2007, 12, 185). Etter en HP eradikering vil slimhinnebetennelsen gradvis forsvinne og tilhørende immunrespons bli redusert. I noen tilfeller utvikler betennelsen seg i mer TH2–retning med autoimmun gastritt- som er forløperen til den premaligne tilstanden atrofisk gastritt. Hvorvidt all atrofisk gastritt går via en autoimmun tilstand er ikke avklart.

- 1. *Kronisk Helicobacter pylori infeksjon, immunrespons og kreftutvikling*

Mekanismene bak utviklingen av cancer ventrikuli som assosieres til HP infeksjon er ikke avklart. Man antar derimot at immunapparatet spiller en vesentlig rolle ved både motstanden mot kreftutvikling og kreftframkallende utvikling. Man antar at cancer ventrikuli starter med en muterende celle i ventrikkelmukosa. Hvilke(n) celletyper som muterer er ikke endelig avklart. Vårt immun apparat vil da reagere mot denne fremmede cellen med en TH1 respons. Hypotesen som Gastroenterologisk laboratorium, UiT forsker på er at dersom man får utvikling av kreft ser man en TH2 deviering av immunresponsen enten som en direkte årsak til eller som en følge av kreftsykdommen (for oversikt, se Cui G and Florholmen J et al Inflammation & Allergy – DrugTarget 2008, 2008, 7:94). Siste års forskning har gitt nye immunologiske fenotyper som assosieres til HP assosiert cancer ventrikuli. Shuiping T og medforfatter har vist i en transgenisk musemodell at HP induserer ventrikkel cancer via en interleukin (Il)-1 beta mekanisme (Cancer Cell 2008, 14, 408). Ulike kliniske studier har foreslått polymorfisme i ulike proinflammatoriske cytokiner slik som Il-1beta, TNF alfa, og Il-6 (for oversikt, se Shuiping T). Det nylige oppdagede Il-33 (Th2 familien) cytokinet finnes normalt i vaskulære epitelceller men forsvinner ved kreftutvikling (Küchler AM et al 2008, Am J Pathol 173, 1229). Videre er TNFR1/Il-17/Il-23 assosiert til ovarial kreft utvikling i en dyremodell (Charles KE et al. J Clin Invest 2009, 119, 3011) og polymorfisme i Il-17 genet er assosiert til gastrisk carcinogenese (Shibata T et al. Hum Immunol 2009, 70, 547).

*1.5. Bakgrunn og målsetning ved studien.*

Samspillet mellom bakterie og vert ved kronisk HP infeksjon er komplekst, Muligens kan en del immunologiske mekanismer forklare immunologiske fenotyper slik som antibiotika resistens og/eller kreftutvikling. Man vil med denne studien forsøke å belyse om det er immunologiske faktorer i mageslimhinnen som kan gi økt malignitetspotensiale.

Man vil også forsøke å belyse immunologiske forhold i vertens mageslimhinne og forhold ved bakterien som kan gi forklaringer på manglende effekt av eradikasjonsbehandling.

Man vil se på resistensforhold ved bakterien og se om det har vært lignende utvikling av resistens i Norge.

## MATERIALE OG METODER

**Materiale og forøksdesign- se ellers Flyteskjema**

Pasientmateriale består av ett hovedmateriale samt 5 kontroll grupper

1. Hovedmaterialet: 40 pasienter fra UNN som har blitt behandlet for sykdom i øvre GI-traktus forårsaket av HP-infeksjon funnet ved gastroskopi og påvist ved urease hurtigtest i tiden 1/1-96 -31/12-2002. Disse pasienter har fått behandling mot HP infeksjonen. De som fortsatt er HP-positve etter 2 behandlinger med antibiotika defineres som HP-behandlingsresistente. Disse 40 pasienter får en forespørsel om å delta i studien - man forventer et antall på 30 pasienter som er villig til å delta. i den hensikt å:

- Karakterisere klinisk fenotype: Ulcus ventriculi, ulcus duodeni, gastritt, kjønn, alder
- Gastroskopi med biopsitaking for analyse histologisk, mikrobiologisk og immunologisk
- Tilbud om ny behandling etter resistensbestemmelse
- Kontrollgastroskopi med biopsier etter 3 mndr. for histologiske, bakteriologiske og immunologiske analyser

1. Kontrollgrupper på hver 30 pasienter (gjelder både Vestre Viken HF Ringerike sykehus og UNN unntatt gruppe D).:

- A) Tidligere (> 5 år) HP-eradikerte. Disse rekrutteres fra henvisninger til gastroskopi der det kommer fram at de tidligere har fått erdadikert HP infeksjonen. Disse tilbys gastroskopi med biopsitaking for histologisk, bakteriologisk og immunologisk analyse. Klinisk fenotyping.
- B) Nydiagnostiserte HP-positive ulcus ventriculi, ulcus duodeni og erosiv gasrtritt. Gastroskopi med biopsitaking for histologisk, bakteriologisk og immunologisk analyse. Tilbys behandling for HP.infeksjon iht rutiner. Kontrollgastroskopi 3 mndr. Senere hvor biopsitaking gjentas
- C) HP-negative uten mukosale forandringer. Gastroskopi med biopsi for histologisk, bakteriologisk og immunologisk analyse.
- D) Personer som ikke har gastrointestinalsykdom. Analyse av tidligere biopsier tatt ved Sørreisaundersøkelsen. Her vil man benytte hele tilgjengelig materiale.
- E) Pasienter med ca.ventriculi og som er HP positive. Gastroskopi for histologisk, bakteriologisk og immunologisk analyse. Her vil antallet nødvendigvis bli færre pga av vanskelig rekruttering av kombinasjonen HP positiv og ca ventrikuli. Vi planlegger derfor 15 pasienter.

1. Bakteriologiske analyser:

- Påvise bakterien
- Utvidet resistensbestemmelse (amoxicillin, claritromycin, mitronidazol, fluorokinoloner, rifampiner)

1. Histologisk analyser (Patologisk avdeling UNN/ patologisk avdeling, Sykehuset Buskerud

- Proliferasjonindeks – Ki67
- Sydneyklassifisering

1. Immunologiske analyser:

- Immunhistokjemi
- PCR-analyser av cytokiner
- Proteinanalyser av cytokiner
- Enkeltcelle analyser (på utvalgte UNN pasienter).

Ved Laboratorium for Gastroenterologi og Ernæring har man følgende tilgjengelige metoder til undersøkelse av biopsier: :

“Real time” kvantitativ måling av mRNA (RT-PCR) (i leverbiopsier); TNF-alfa, IL1-, IFN-gamma, IL-4, IL-6, Il-8, IL-12, Il-17, Il-23, Il-33, transkripsjonsfaktorene TBX21, GATA3, and ROR-gamma og BCL-2, Bax. HP assosiert Cag A og Vac A.Immunohistokjemiske teknikker for påvisning av forskjellige cytokiner, apoptose og ulike immunaktive celler (CD4, dendritt-celler, makrofager, aktiverte fibroblaster). Magnetisk assistert cellesortering (MACS) for funksjonelle analyser av celler isolert fra ventrikkelbiopsier

*Statistikk.* Man benytter tilsvarende statistiske metoder som i de kliniske og molekylærbiologske publiserte artikler, se CV Jon Florholmen

**Statistikk**

Man vil benytte ulike statistiske metoder til vurdering av kliniske og laboratoriedata- dette framkommer i tidligere publikasjoner til dr med Rasmus Goll og dr med Anne Mette Asfeldt- se under publikasjonslisten til Jon Florholmen. Det dreier seg om student T test, Wilcoxon sum rank test, Kruskal-Walls test, Mann-Whitney test med Bonferroni korreksjoner og Spearman’s korrelasjons test.

**Godkjenninger**

Det søkes godkjenning fra Regionaletisk komite (Nord Norge) mens biologisk databank er allerede opprettet (HP studien til dr med Rasmus Goll).

## Status oktober 2009

Innsamlet data for 7 års materiale i tiden 1/1-96 – 31/12-02 som er hentet fra journalsystemet på UNN. Det er pasienter som enten har vært innlagt eller vært til poliklinisk behandling og som har fått diagnosene K25 – K29 i diagnosesystemet ICD10 eller 531.0 – 533.1 i ICD9 og som i de aller fleste tilfeller har fått utført gastroskopi (unntaksvis noen som har fått diagnosen ved pusteprøve). Det er registrert 40 pasienter.

Man har registrert kjønn, alder på pasient, type kur der det har vært mulig å finne, evt. flere kurer og om pasienten er eradikert.

3. PROSJEKTER

Følgende delprosjekter vil bli gjennomført og evaluert:

*3.1 Kliniske og mikrobiologiske fenotyper av HP behandlingsresistente pasienter.*

Her vil man innkalle de 40 registrerte HP behandlingsresistente pasientene som har hatt sykdommen i 12-15 åretter mislykket HP eradikering. Man vil karakterisere alder, kjønn, ulcus sykdom tidligere (før initiell behandling) og nå, årlig behov for antall syrereduserende kurer, *quality of life* registreringer samt effekt av ny eradikeringsbehandling.

HP bakterien vil bli karakterisert som antibiotika resistens mønster samt påvisning av toxiner (Cag A og Vac A) vil bli bestemt.

Dette er en original studie da de kliniske og mikrobiologiske fenotyper ikke er kartlagt for behandlingsresistente HP positive pasienter.

Planlagt artikkel: *Nestegard O et al. Clinical and microbiological characterizations of Helicobacter pylori P infected patients 15 years after unsuccessful eradication*

*3.2. Immunologisk karakterisering av ventrikkel mukosa etter langvarig HP infeksjon hos behandlingsresistente pasienter.*

Hypotesen er at det gjennom en langvarig HP infeksjon slik vår gruppe av HP behandlingsresistente pasienter representerer, vil immunresponsen i ventrikkel mukosa endre seg til et premalign immunofenotypisk mønster.

Valget av kontrollgrupper (gruppe B-E, se over) gjør en slik vurdering mulig. ”Normal” kontrollen vil være HP negative pasienter uten endoskopiske forandringer i øvre GI traktus HP eradikerte pasienter > 5 år etter eradikeringen vil være en viktig sammenlignbar kontroll. Kontrollgruppen nylig påvist sykdom med påfølgende eradikering vil vi velge pasienter < 50 år for å utelate de med antatt langvarig HP infeksjon. Dette er ingen ideell kontroll da vi ikke vet hvor lenge de har hatt sykdommen. Disse pasienter vil bli HP eradikert og mukosa immunologien før og etter behandlingen vil gi oss nyttig kunnskap i den totale vurderingen av mukosa immunologien i et langvarig HP infeksjon. HP infisert ventrikkel mukosa hos pasienten med magekreft er også en viktig kontroll gruppe i egenskap av at i denne mukosaen er det etablert malign sykdom.

Dette materialet vil bli gjengang for en deskriptiv immunologisk og bakteriologisk karakterisering. Deretter vil man iht funnene gjøre mer spesifikke analyser for å se på mekanismene bak utviklingen av sykdommen.

Man vil planlegge to hovedartikler:

Nestegard O et al. Immunological phenotypes in gastric mucosa after longstanding HP infection.

Nestegard O et al. Helicobacter pylori resistance profiles and toxins in treatment sensitive an insensitive patients.

Man vil iht til overnevnte funn gjøre mere mekanistiske analyser i perspektiv av malign utvikling på bakgrunn av en langvarig HP infeksjon.

4. FRAMDRIFTSPLAN

3 års prosjekt. Innsamling av data vil skje fra 1 januar 2010 til 3112 2011. Manuskript bearbeidelse vil starte allerede i 2010 og forventes avsluttes 31122012.

5 . BUDSJETT

Utgifter til prosjektet er i hovedsak utgifter til primærforsker (overlege Oddmund Nestegard). Han vil trenge en 20 % stilling de to første årene og en 100 % stilling siste del av forskningsperioden. Disse utgiftene vil man søke lokalt (Ringerike) samt HelseNord.

Analyseutgiftene vil bli dekket av Gastrofondet UNN.

6. BETYDNING

Dette prosjektet vil gi ny kunnskap på 2 hovedområder: ny kunnskap om konsekvenser av langvarig kronisk HP infeksjon som både nasjonalt men særlig globalt er et stort klinisk problem; hvilken betydning en kronisk HP infeksjon har for utvikling av kreftsykdom. Denne assosiasjonen er vel dokumentert. Så langt har man vegret seg for å fjerne bakterien pga av bl.a. at store deler av befolkningen er smittet i enkelte deler av verden. Man må derfor måtte konsentrere seg om å behandle spesielle utsatte grupper. Denne studien vil kunne gi mer kunnskap om det finnes spesielle immunologiske fenotyper som er i en risikogruppe for kreftutvikling.
