# Supplementary material for: Helicobacter pylori resistance to antibiotics before and after treatment: Incidence of eradication failure
Source: PLoS One. 2022 Apr 20;17(4):e0265322. doi: 10.1371/journal.pone.0265322 (PMC9020706; doi:10.1371/journal.pone.0265322)
Supplement: S1 Table — (DOCX) [file pone.0265322.s003.docx]

| Number Amoxicillin |  |
| --- | --- |
| Ciprofloxacin | Total |
| 0,032 | 4 |
| 0,064 | 20 |
| 0,125 | 26 |
| 0,25 | 13 |
| 0,5 | 5 |
| 1 | 1 |
| > 32 | 3 |
| (empty) | 5 |
| Total | 77 |

| Number Amoxicillin |  |
| --- | --- |
| Levofloxacin | Total |
| 0,032 | 1 |
| 0,064 | 18 |
| 0,125 | 32 |
| 0,25 | 15 |
| 0,5 | 3 |
| 1 | 1 |
| 4 | 1 |
| 16 | 1 |
| (empty) | 5 |
| Total | 77 |

| Number Amoxicillin |  |
| --- | --- |
| Ofloxacin | Total |
| 0,125 | 13 |
| 0,25 | 22 |
| 0,5 | 24 |
| 1 | 9 |
| 2 | 2 |
| 8 | 1 |
| 16 | 1 |
| (empty) | 5 |
| Total | 77 |

| Number Amoxicillin |  |
| --- | --- |
| Nalidixinacid | Total |
| > 256 | 72 |
| (empty) | 5 |
| Total | 77 |

| Number Amoxicillin |  |
| --- | --- |
| Moxifloxacin | Total |
| 0,016 | 1 |
| 0,032 | 3 |
| 0,064 | 19 |
| 0,125 | 39 |
| 0,25 | 6 |
| 0,5 | 1 |
| 4 | 2 |
| 16 | 1 |
| (empty) | 5 |
| Total | 77 |

| *Number Amoxicillin* | Levofloxacin |  |  |  |  |  |  |  |  |  |
| --- | --- | --- | --- | --- | --- | --- | --- | --- | --- | --- |
| Ciprofloxacin | 0,032 | 0,064 | 0,125 | 0,25 | 0,5 | 1 | 4 | 16 | (empty) | Total |
| 0,032 | 1 | 3 |  |  |  |  |  |  |  | 4 |
| 0,064 |  | 12 | 7 | 1 |  |  |  |  |  | 20 |
| 0,125 |  | 3 | 18 | 4 | 1 |  |  |  |  | 26 |
| 0,25 |  |  | 5 | 8 |  |  |  |  |  | 13 |
| 0,5 |  |  | 2 | 2 | 1 |  |  |  |  | 5 |
| 1 |  |  |  |  | 1 |  |  |  |  | 1 |
| > 32 |  |  |  |  |  | 1 | 1 | 1 |  | 3 |
| (empty) |  |  |  |  |  |  |  |  | 5 | 5 |
| Total | 1 | 18 | 32 | 15 | 3 | 1 | 1 | 1 | 5 | 77 |

| Number Amoxicillin |  |
| --- | --- |
| Metronidzole | Total |
| 0,032 | 1 |
| 0,064 | 5 |
| 0,125 | 6 |
| 0,25 | 12 |
| 0,5 | 8 |
| 1 | 1 |
| 2 | 3 |
| 4 | 2 |
| 8 | 1 |
| 12 | 1 |
| 32 | 2 |
| 192 | 1 |
| < 0,016 | 1 |
| > 256 | 33 |
| Total | 77 |

| Number Amoxicillin |  |
| --- | --- |
| Amoxicillin | Total |
| 0,032 | 6 |
| 0,064 | 6 |
| 0,125 | 3 |
| < 0,016 | 62 |
| Total | 77 |

| Number Amoxicillin |  |
| --- | --- |
| Tetracycline | Total |
| 0,032 | 1 |
| 0,064 | 3 |
| 0,125 | 16 |
| 0,25 | 24 |
| 0,5 | 22 |
| 1 | 11 |
| Total | 77 |

| Number Amoxicillin |  |
| --- | --- |
| Clarithromycin | Total |
| 0,032 | 1 |
| 0,064 | 1 |
| 0,125 | 1 |
| 0,5 | 1 |
| 1 | 7 |
| 2 | 2 |
| 4 | 1 |
| 64 | 1 |
| 128 | 1 |
| < 0,016 | 61 |
| Total | 77 |
