# Supplementary material for: Helicobacter pylori resistance to antibiotics before and after treatment: Incidence of eradication failure
Source: PLoS One. 2022 Apr 20;17(4):e0265322. doi: 10.1371/journal.pone.0265322 (PMC9020706; doi:10.1371/journal.pone.0265322)
Supplement: Attachment — Submitted filename: PONE-D.docx [file pone.0265322.s005.docx]

PLOS ONE

# Helicobacter pylori resistance to antibiotics in patients 15 years after unsuccessful eradication

--Manuscript Draft--

| **Manuscript Number:** | PONE-D-21-21216 |
| --- | --- |
| **Article Type:** | Research Article |
| **Full Title:** | Helicobacter pylori resistance to antibiotics in patients 15 years after unsuccessful eradication |
| **Short Title:** | Resistance to Helicobacter pylori. |
| **Corresponding Author:** | Oddmund Nestegard, M.D. Vestre Viken HF Hønefoss, NORWAY |
| **Keywords:** | Amoxicillin; clarithromycin; levofloxacin; tetracycline |
| **Abstract:** | Background  Increasing prevalence of antibiotic resistance especially to clarithromycin and metronidazole has been observed in Helicobacter pylori (H. pylori).  Aim  To characterize the antimicrobial resistance pattern of H. pylori before and after treatment in a cohort of patients accumulated over a period of 15 years after an unsuccessful eradication treatment had been given comparing sensitivity data from patients with newly diagnosed H. pylori infection. A specific objective was to look for resistance to levofloxacin.  Material and Methods  Total of ~~Included were~~ 50 patients newly diagnosed for H. pylori infection treated with omeprazole and amoxicillin/clarithromycin, and 42 H. pylori treatment-resistant patients treated with omeprazole and levofloxacin were enrolled in this study. Cultures including antibiotic sensitivity testing were conducted according to standard laboratory routines and thus also in keeping with a European study protocol using E-test gradient strips or disc diffusion methods.  Results  ~~Before treatment~~ Clarithromycin resistance was more frequently observed in the H. pylori resistant group than in the newly diagnosed *H. pylori* group (39% versus 11%). Regarding metronidazole, the distribution was 70% versus 38%, and 8% versus 12% were resistant to tetracycline. No resistance was observed for amoxicillin.  After re-treatment of patients belonging to the H. pylori treatment-resistant group, just two patient strains were recovered of which one harbored metronidazole resistance.  In the group of newly diagnosed H. pylori, seven patients were culture positive by control after treatment. Two and three patient strains showed resistance to clarithromycin and metronidazole, respectively. None of the strains in our material was classified as resistant to amoxicillin and levofloxacin. Whereas 12% ~~resistance~~ was ~~present~~ resistant to tetracycline in the newly diagnosed before treatement.  Conclusion  ~~Strains recovered from patients after unsuccessful treatment for H. pylori infection showed~~ Clarithromycin resistance was more frequent in the H. pylori resistant group than strains from patients with newly diagnosed H. pylori infection. No resistance was observed to amoxicillin and levofloxacin. ~~In such cases~~ Therefore levofloxacin may be the best choice to eradicate H. pylori ~~used~~ provided in vitro sensitivity testing confirms applicability. |
| **Order of Authors:** | Oddmund Nestegard, M.D. |
|  | Behrouz Moayeri |

*Powered by Editorial Manager® and ProduXion Manager® from Aries Systems Corporation*

|  | Fred-Arne Halvorsen |
| --- | --- |
|  | Tor Tønnesen |
|  | Sveinung Wergeland Sørbye |
|  | Eyvind Paulssen |
|  | Kay-Martin Johnsen |
|  | Rasmus Goll |
|  | Jon Ragnar Florholmen |
|  | Kjetil Melby |
| **Additional Information:** |  |
| **Question** | **Response** |
| **Financial Disclosure**  Enter a financial disclosure statement that describes the sources of funding for the work included in this submission. Review the [submission guidelines](http://journals.plos.org/plosone/s/submission-guidelines#loc-financial-disclosure-statement) for detailed requirements. View published research articles from [*PLOS ONE*](http://journals.plos.org/plosone/) for specific examples.  This statement is required for submission and **will appear in the published article** if the submission is accepted. Please make sure it is accurate. | No.  The study is funded by Research Department, Vestre Viken Hopital, Drammen The funders had no role in study design, data collection and analysis, decision to publish, or preparation of the manuscript |
| **Unfunded studies**  Enter: *The author(s) received no specific funding for this work.*  **Funded studies**  Enter a statement with the following details:   - Initials of the authors who received each award - Grant numbers awarded to each author - The full name of each funder - URL of each funder website - Did the sponsors or funders play any role in the study design, data collection and analysis, decision to publish, or preparation of the manuscript? - **NO** - Include this sentence at the end of your statement: *The funders had no role in study design, data collection and analysis, decision to publish, or preparation of the manuscript.* - **YES** - Specify the role(s) played. |  |
| * typeset |  |
| **Competing Interests** | No |

*Powered by Editorial Manager® and ProduXion Manager® from Aries Systems Corporation*

| Use the instructions below to enter a competing interest statement for this submission. On behalf of all authors, disclose any [competing interests](http://journals.plos.org/plosone/s/competing-interests) that could be perceived to bias this work—acknowledging all financial support and any other relevant financial or non- financial competing interests.  This statement is required for submission and **will appear in the published article** if the submission is accepted. Please make sure it is accurate and that any funding sources listed in your Funding Information later in the submission form are also declared in your Financial Disclosure statement.  View published research articles from  [*PLOS ONE*](http://journals.plos.org/plosone/) for specific examples. |  |
| --- | --- |
| **NO authors have competing interests**  Enter: *The authors have declared that no competing interests exist.*  **Authors with competing interests**  Enter competing interest details beginning with this statement:  *I have read the journal's policy and the authors of this manuscript have the following competing interests: [insert competing interests here]* |  |
| * typeset |  |
| **Ethics Statement**  Enter an ethics statement for this submission. This statement is required if the study involved:   - Human participants - Human specimens or tissue - Vertebrate animals or cephalopods - Vertebrate embryos or tissues | The study is approved by the Regional Committee for Medical and Health Research Ethics (REC North ID:2009/2176-15) including approved storage of biological material. Consents are obtained in written form. |

*Powered by Editorial Manager® and ProduXion Manager® from Aries Systems Corporation*

| - Field research   Write "N/A" if the submission does not require an ethics statement.  General guidance is provided below. Consult the [submission guidelines](http://journals.plos.org/plosone/s/submission-guidelines#loc-guidelines-for-specific-study-types) for detailed instructions. **Make sure that all information entered here is included in the Methods section of the manuscript.** |  |
| --- | --- |

*Powered by Editorial Manager® and ProduXion Manager® from Aries Systems Corporation*

| **Format for specific study types**  **Human Subject Research (involving human participants and/or tissue)**   - Give the name of the institutional review board or ethics committee that approved the study - Include the approval number and/or a statement indicating approval of this research - Indicate the form of consent obtained (written/oral) or the reason that consent was not obtained (e.g. the data were analyzed anonymously)   **Animal Research (involving vertebrate animals, embryos or tissues)**   - Provide the name of the Institutional Animal Care and Use Committee (IACUC) or other relevant ethics board that reviewed the study protocol, and indicate whether they approved this research or granted a formal waiver of ethical approval - Include an approval number if one was obtained - If the study involved *non-human primates*, add *additional details* about animal welfare and steps taken to ameliorate suffering - If anesthesia, euthanasia, or any kind of animal sacrifice is part of the study, include briefly which substances and/or methods were applied   **Field Research**  Include the following details if this study involves the collection of plant, animal, or other materials from a natural setting:   - Field permit number - Name of the institution or relevant body that granted permission |  |
| --- | --- |
|  |  |
| **Data Availability**  Authors are required to make all data underlying the findings described fully available, without restriction, and from the time of publication. PLOS allows rare exceptions to address legal and ethical concerns. See the [PLOS Data Policy](http://journals.plos.org/plosone/s/data-availability) and [FAQ](http://journals.plos.org/plosone/s/data-availability#loc-faqs-for-data-policy) for detailed information. | Yes - all data are fully available without restriction |

*Powered by Editorial Manager® and ProduXion Manager® from Aries Systems Corporation*

| A Data Availability Statement describing where the data can be found is required at submission. Your answers to this question constitute the Data Availability Statement and **will be published in the article**, if accepted. |  |
| --- | --- |
| **Important:** Stating ‘data available on request from the author’ is not sufficient. If your data are only available upon request, select ‘No’ for the first question and explain your exceptional situation in the text box. |  |
| Do the authors confirm that all data underlying the findings described in their manuscript are fully available without restriction? |  |
| **Describe where the data may be found in full sentences. If you are copying our sample text, replace any instances of XXX with the appropriate details.**   - If the data are **held or will be held in a public repository**, include URLs, accession numbers or DOIs. If this information will only be available after acceptance, indicate this by ticking the box below. For example: *All XXX files are available from the XXX database (accession number(s) XXX, XXX.*). - If the data are all contained **within the manuscript and/or Supporting Information files**, enter the following: *All relevant data are within the manuscript and its Supporting Information files.* - If neither of these applies but you are able to provide **details of access elsewhere**, with or without limitations, please do so. For example:   *Data cannot be shared publicly because of [XXX]. Data are available from the XXX Institutional Data Access / Ethics Committee (contact via XXX) for researchers who meet the criteria for access to confidential data.*  *The data underlying the results presented in the study are available from (include the name of the third party* | Data are available from the AiT-Artic University Norway (contact prof. Jon Florholmen, jon.florholmen@unn, for researchers who meet the criteria for access to confidential data. |

*Powered by Editorial Manager® and ProduXion Manager® from Aries Systems Corporation*

| *and contact information or URL).*   - This text is appropriate if the data are owned by a third party and authors do not have permission to share the data.   * typeset |  |
| --- | --- |
| Additional data availability information: | Tick here if the URLs/accession numbers/DOIs will be available only after acceptance of the manuscript for publication so that we can ensure their inclusion before publication.; Tick here if your circumstances are not covered by the questions above and you need the journal’s help to make your data available. |

*Powered by Editorial Manager® and ProduXion Manager® from Aries Systems Corporation*

Manuscript	[Click here to access/download;Manuscript;Resistens 110821 (3).docx](https://www.editorialmanager.com/pone/download.aspx?id=29708234&guid=89de7dd3-8cde-45ae-ba09-f16910197108&scheme=1)

# Helicobacter pylori resistance to antibiotics in patients 15 years after unsuccessful eradication

**ClinicalTrials.gov Identifier: NCT05019586**

Nestegard O^1,2^, Moayeri B^3^, Halvorsen FA^4^, Tønnesen T^4^, Sørbye SW^5^, Paulssen, EJ^2^, Kay- Martin Johnsen^2^, Goll R^2^, Florholmen J^2^, Melby KK^3,6^,

*^1^Ringerike Hospital, Vestre Viken HF, ^2^ Research Group Gastroenterology and Nutrition, Department of Clinical Medicine, UiT the Artic University Norway, ^3^ Department of Microbiology, Oslo University Hospital, ^4^Drammen Hospital, Vestre Viken Hospital Trust, ^5^Department of Pathology, University Hospital of North Norway, Tromsø. ^6^Institute for Clinical Medicine, Faculty of Medicine, University of Oslo.*

#### ABSTRACT

**Background**

Increasing prevalence of antibiotic resistance especially to clarithromycin and metronidazole has been observed in *Helicobacter pylori (H. pylori).*

#### Aim

To characterize the antimicrobial resistance pattern of *H. pylori* before and after treatment in a cohort of patients accumulated over a period of 15 years after an unsuccessful eradication treatment had been given comparing sensitivity data from patients with newly diagnosed *H. pylori* infection. A specific objective was to look for resistance to levofloxacin.

**Material and Methods**

Included were 42 treatment-resistant *H pylori* patients treated with omeprazole and levofloxacin and 50 patients with newly diagnosed *H. pylori* infection treated with omeprazol and amoxicillin/clarithromycin. Cultures including antibiotic sensitivity testing were conducted according to standard laboratory routines and thus also in keeping with a European study protocol using E-test gradient strips or disc diffusion methods.

**Results**

Before treatment clarithromycin resistance was more frequently observed in the H. pylori

resistant group than in newly diagnosed *H. pylori* group (39% versus 11%), for

metronidazole the distribution was 70% versus 38%, and 8% versus 12% were resistant to tetracycline. No resistance was observed for amoxicillin. After re-treatment of patients belonging to the *H. pylori* treatment resistant group, just two patient strains were recovered of which one harbored metronidazole resistance. In the group of newly diagnosed *H. pylori,* seven patients were culture positive by control after treatment. Two and three patient strains showing resistance to clarithromycin and metronidazole, respectively. None of the strains in

our material was classified as resistant to amoxicillin, levofloxacin, whereas 12% resistance was present to tetracycline in the newly diagnosed.

#### Conclusion

Strains recovered from patients after unsuccessful treatment for *H. pylori* infection showed clarithromycin resistance more frequently than strains from patients with newly diagnosed *H. pylori* infection. No resistance was observed to amoxicillin and levofloxacin. In such cases levofloxacin may be used provided in vitro sensitivity testing confirms applicability.

**Running title:** Helicobacter pylori resistance Guarantor of the article: Oddmund Nestegard

Declaration of interest: none

All authors have approved ~~of~~ the final version for publication.

Keywords: ~~amoxicillin, clarithromycin, levofloxacin, tetracycline~~ Helicobacter pylori, eradication, antibiotics,

resistance

Address for correspondence Oddmund Nestegard, mail: [oddmuan@online.no](mailto:oddmuan@online.no)

Phone number +47 90730595

#### Introduction

*Helicobacter pylori* (*H. pylori*) was discovered in 1983 [1] and has been identified as the main pathogenic factor for gastric and duodenal peptic ulcers [2]. Treatment of the *H. pylori* infection cures patients with such ulcers, thus this disease is no longer a chronically recurring and disabling condition in the Western world. However, ~~Still~~ the infection is still a great problem worldwide, especially in developing countries, where up to 50% of the population may be infected [3-4].

Initially, the most effective eradication regimens had an efficacy of >90% [5]. Unfortunately, after 20 years of infection treatment, t~~here has been an~~ increasing antimicrobial resistance of *H. pylori* has been installed [6]. This includes resistance to clarithromycin and metronidazole ~~and has been proposed to be a consequence of~~ due to the increased use of these antibiotics for other conditions.

Clarithromycin resistance has especially had a major negative impact as it has been a main constituent of the recommended first-line triple therapy for *H. pylori* eradication.

Metronidazole resistance is highly prevalent but can to some extent be overcome [7]. Thus, the increasing prevalence of antibiotic resistance has to be considered when designing rational therapeutic regimens for *H. pylori* infection [8-9]. In areas with a known high degree of resistance to both clarithromycin and metronidazole, bismuth quadruple therapy is recommended [10].

Parallel to the increasing antibiotic resistance, a cohort of patients infected with treatment-resistant *H. pylori* has accumulated. These patients will be at risk for the recurrence of peptic ulcer disease. Few reports exist on the clinical or ~~the~~ microbial characterizations of this cohort of patients. However, some data are emerging in the search for a new effective antibiotic therapy, including levofloxacin [11]. Thus, the goal of this study was to characterize the antimicrobial resistance pattern of *H. pylori* in a cohort of patients accumulated over a period of 15 years after an unsuccessful eradication attempt.

#### Materials and Methods

***Study population***

*Enrollment and patient flow*

The data presented are from the main Chronical Infection of Helicobacter Pylori (CHRIHEP)

study published elsewhere [12]. ~~In short~~, patients were recruited from three different cohorts admitted to three different gastrointestinal units at Norwegian hospitals: i.e. The University Hospital of North Norway, Tromsø; Drammen Hospital, Drammen and Ringerike Hospital, Hønefoss, the latter two both being parts of Vestre Viken Hospital Trust. We report the data obtained from the two groups of interest with active *H. pylori* infection:

Group 1, *(H. pylori* treatment-resistant n=42), were recruited from medical files from 1990

to 2012, and from patients that had been treated unsuccessfully two or more times for *H. pylori* infection with different regimens [12]. All persons who completed treatment and controls.

Group 2 (*H. pylori* untreated, n=50) were outpatients referred to the Gastrointestinal unit for upper endoscopy due to gastrointestinal complaints and diagnosed as being *H. pylori-*infected for the first time [12]. There were 52 patients, two of them denied participation.

The diagnosis of *H. pylori* in biopsies was based on a positive result for either the *H. pylori* rapid urease test or detection of the presence of *H. pylori* in histological specimens, or both [12].

*Determination of antimicrobial sensitivity.*

Bacterial strains, isolated between 2010 and 2016, were examined using a standardized technique according to a European study protocol [9]. All isolates were recovered from biopsies from the stomach lining in patients with suspected *H. pylori*-related disease.

Identification was based on colony morphology, Gram staining**,** and positive catalase, oxidase, and urease tests. The strains were kept at -70ºC. The minimum inhibitory concentrations (MICs) of metronidazole, amoxicillin, clarithromycin, tetracycline and levofloxacin were determined by E-test (bioMerieux, France). Levofloxacin representing the quinolone group of antibiotics. The inoculum was adjusted to McFarland standard 3. Mueller Hinton agar (Beckton Dickinson) with 10% horse blood was used. Agar plates were incubated for 72-96 hours in microaerobic conditions (Campygen, OXOID, UK). MIC values were read according to the instructions from the manufacturer.

*Treatment*

Patients in Group 1 (*H. pylori* resistant) have ~~then~~ retreated with oral omeprazole 20 mg b.i.d., amoxicillin 1 g b.i.d, and levofloxacin 500 mg b.i.d. for 10 days. Six of the 42 patients were still *H. pylori-*positive after treatment. In Group 2 (*H. pylori* naïve), ten of 50 patients (20%) were *H. pylori-*positive after treatment of oral omeprazole 20 mg b.i.d, amoxicillin 1 g b.i.d., and clarithromycin 500 mg b.i.d. for 7 days [12]. Subjects in both groups underwent gastroscopy 3-6 months after treatment and biopsies were collected for the various examinations described above

#### Results

A total of 92 patients, divided into 42 in Group 1 and 50 in Group 2, were enrolled according to the inclusion and exclusion criteria. Results from bacterial cultures of gastric mucosal biopsies are shown in Table 1. Among patients with positive *H. pylori* tests, positive cultures were obtained from 26 of 42 and 42 of 50 patients in Group 1 and Group 2, respectively, before treatment in this study (visit 1). After treatment (visit 2), two of ~~47~~ 37 and nine of 42 cultures were positive for *H. pylori*, respectively (Table 1). Two of the nine positive cultures in Group 2 did not yield valid results. In Table 2 the detailed antimicrobial resistance data are shown both before (Visit 1) and after treatment (Visit 2).

At visit 1, in Group 1 clarithromycin resistance was more frequently observed than in Group 2 (39% versus 11%), for metronidazole the distribution was 70% versus 38%, for tetracycline

the distribution was 8% versus 12%. No resistance was observed for amoxicillin.

At visit 2, in Group 1 only two cultures were positive, where one culture showed metronidazole resistance. At visit 2, in Group 2, seven cultures were positive, two and three with resistance for clarithromycin and metronidazole, respectively.

None of the strains in our material was classified as resistant to amoxicillin or levofloxacin, whereas some resistance was present to tetracycline.

#### Discussion

In the cohort of patients with treatment failures (secondary resistance) to *H. pylori*,

accumulated over 15 years, the most prevalent resistance pattern was observed against metronidazole (70%) and a somewhat lower prevalence against clarithromycin (39%), even less against tetracycline (8%) and none towards amoxicillin. These data are in keeping with data presented from other research groups and general surveillance of antibiotic sensitivity of *H. pylori* [13]. The ~~corresponding~~ prevalence of antibiotic resistance in newly diagnosed patients for *H. pylori* infection (primary resistance) was ~~11~~% 38% for metronidazole and ~~38~~% 11% for clarithromycin, half of the numbers for secondary resistance. None of the strains in our study was classified as resistant to neither amoxicillin nor levofloxacin. Even with these antibiotic resistance patterns, we achieve eradication rates of 79% and 92% in the Hp naïve and Hp resistant groups, respectively. Thus, our study indicates that secondary *H. pylori* resistance to metronidazole and clarithromycin may be overcome by the application of new antibiotic combinations. Over the last years, reports from different parts of the world have been published dealing with antimicrobial resistance rates in *H. pylori* [14]. At present, WHO considers *H. pylori* to be an important threat to human health based on the global wide

appearance and declining antibiotic sensitivity [15]. Comparing results from different reports may, however, be difficult as in many cases different methodologies have been applied [14]. In addition, some presentations do not specify whether the reported numbers reflect primary resistance alone or a mix of strains from both categories [14].

Furthermore, higher levels of *H. pylori* primary antibiotic resistance might be expected in general in countries where antibiotic usage is higher than for instance in the Nordic countries (ECDC report 2015). [16] A European multicenter study reported in 1997 an average primary resistance rate in Europe of 9.9% to clarithromycin [17]. Another study from 2013 showed that resistance rates to many drugs currently in use correlated with local antibiotic consumption [18]. In this study, the primary resistance to clarithromycin varied between 5.6% and 36.6%. An analysis in 2019 on the antibiotic sensitivity pattern in *H. pylori* in South-Eastern Norway showed a clarithromycin resistance of < 10% [19]. This figure corresponds with data obtained on clarithromycin use in the same period [20]. These findings support the lesser occurrence of antibiotic resistance in *H. pylori* provided a corresponding restricted prescription of clarithromycin. A follow-up study on the relationship between antibiotic consumption and *H. pylori* resistance support this notion [13].

There is no indication of increasing primary resistance to clarithromycin in our catchment area over the last 10 years as the present study showed an MIC50 value (the MIC value that inhibits 50% of the isolates) of 0.023 for clarithromycin, compared to 0.047 (In- house data, N=23) obtained approximately 10 years ago. Data on primary resistance in Europe was shown to be 24% for clarithromycin, 34% for metronidazole, and 20% for levofloxacin [21].

The reported resistance to metronidazole varies globally. In Europe, the prevalence of metronidazole resistance in *H. pylori* is generally between 20 and 40% [10], whereas the prevalence in developing countries is known to be higher (50-80%) [22]. Differences may

reflect the widespread use of metronidazole in these areas, methodological differences, or weak reproducibility of the analysis. In our study, 69.5% (Group 1) and 38.1 % (Group 2) of the isolates were resistant to metronidazole when the adopted European methodology was applied, i.e., microaerobic conditions for the entire incubation period. However, as metronidazole requires anaerobic conditions to be activated, the Norwegian reference group for antibiotics has recommended that the application of anaerobic conditions be limited to the initial 24 hours of the incubation period when performing susceptibility testing for metronidazole. Applying this approach, the resistance rate towards metronidazole fell from 22.5% to 7.8% in a recent study [9], indicating that a substantial number of the resistant strains might be clinically susceptible to metronidazole. The previously mentioned study from 2013 showed that the primary resistance rate to metronidazole [17] varied between 28.6% and 43.8%. Thus, in our setting the resistance to antibiotics used for the treatment of *H. pylori* is low with the exception of metronidazole and partly clarithromycin. In this context, the situation described from Iceland. Moreover, *H. pylori* resistance to clarithromycin has been reported from other countries leaving the use of levofloxacin for primary treatment resistant cases [23]. Levofloxacin resistance is due to changes in gyrA component of *H. pylori* [21-24] that renders even fluoroquinolones inactive to suppress *H. pylori* growth [21- 24-25].

Based on generally available international data on antibiotic resistance in *H. pylori,* the level of sensitivity seems to vary internationally according to the general use of antibiotics and the antibiotic combinations applied in *H. pylori* therapy [25]. In addition, changes in the antibiotic sensitivity pattern of *H. pylori* may also serve as a sign of antibiotic interference with the human microflora. Use of these drugs should be carefully monitored. Increasing resistance should advocate reduced consumption and to target the use of antibiotics carefully to secure a successful outcome while preserving these valuable drugs for serious infections to

come. Thus, continuous surveillance of antibiotic resistance in this *H. pylori* is warranted in order to secure optimal treatment options for gastric diseases patients and infectious disease patients in general. Finally, the data from the European surveillance study [21-26] indicate an increase in levofloxacin resistance is to be expected and the drug should be reserved primarily for treatment failures. A susceptibility check of the offending strain should definitely be performed to ensure *H. pylori* susceptibility to the drugs to be used for eradicating the bacteria even though such sensitivity testing is, for various reasons, done to a lesser extent than it should be [27].

The strength of this study is we have studied antibiotic resistance in *H. pylori* comparing a previous treatment-resistant group to a group ~~with~~ newly diagnosed for *H. pylori* infection.

Moreover, the potential of resistance to levofloxacin – the apparent drug of choice in treatment-resistant cases- has been studied. The weakness of the study is the lack of ~~a~~ continuous surveillance of antibiotic resistance in this microbe as indicated above. Therefore, a future ongoing study to secure optimal treatment options is highly warranted and especially focuses on levofloxacin resistance.

In conclusion, clarithromycin resistance was more frequently observed in patients resistant to

*H. pylori* treatment than in patients ~~with~~ newly diagnosed for *H. pylori* infection. None of the samples showed resistance to amoxicillin and levofloxacin. This implies that levofloxacin should be the drug of choice in cases resistant to ordinary triple regimens. When antibiotic	therapy is planned, sensitivity testing of the offending strain should be performed to define	the antibiotics to be used and thus increase the chance for successful eradication of the *H. pylori* infection

#### Acknowledgments

We thank Odd Sverre Moen at Research Group Gastroenterology and Nutrition, UiT The Arctic University of Norway, Tromsø, Norway for expert technical assistance.

#### References

1 Warren JR, Marshall BJ. Unidentified curved bacilli on gastric epithelium in active chronic gastritis. Lancet 1983;321:1273–5.
2 Nomura A, Stemmermann GN, Chyou PH, Perez-Perez GI, Blaser MJ. Helicobacter pylori infection and the risk for duodenal and gastric ulceration. Ann Intern Med 1994 Jun 15;120(12):977-81.
3 Go MF. Review article: natural history and epidemiology of Helicobacter pylori infection. Aliment Pharmacol Ther 2002; 16(Suppl 1):3–15.
4 Breckan RK, Paulssen EJ Asfeldt AM, Kvamme JM, Straume B, Florholmen J, The All- Age Prevalence of Helicobacter pylori Infection and Potential Transmission Routes. A Population-Based Study Helicobacter ISSN 1523-5378
5 Lind T, Veldhuyzen van Zanten S, Unge P, Spiller R, Bayerdörffer E, O’Morain C, Bardhan KD, Bradette M, Chiba N, Wrangstadh M, Cederberg C, Idström JP. Eradication of Helicobacter pylori using one-week triple therapies combining omeprazole with two antimicrobials: the MACH I Study. Helicobacter 1996; 1: 138-144 [PMID: 9398894]
6 Megraud F, Lehours P. Helicobacter pylori detection and antimicrobial susceptibility testing. Clin Microbiol Rev 2007;20:280e322.
7 Malfertheiner P, Megraud F, O’Morain C, et al. Current concepts in the management of Helicobacter pylori infection: the Maastricht III Consensus Report.Gut 2007;56:772, 772-81.
8 Fischbach L, Evans EL. Meta-analysis: the effect of antibiotic resistance status on the efficacy of triple and quadruple first-line therapies for Helicobacter pylori. Aliment Pharmacol Ther 2007;26:343e57.
9 Larsen AL, [Ragnhildstveit E](https://www.ncbi.nlm.nih.gov/pubmed/?term=Ragnhildstveit%20E%5BAuthor%5D&cauthor=true&cauthor_uid=23083455), [Moayeri B,](https://www.ncbi.nlm.nih.gov/pubmed/?term=Moayeri%20B%5BAuthor%5D&cauthor=true&cauthor_uid=23083455) [Eliassen L,](https://www.ncbi.nlm.nih.gov/pubmed/?term=Eliassen%20L%5BAuthor%5D&cauthor=true&cauthor_uid=23083455) [Melby KK.](https://www.ncbi.nlm.nih.gov/pubmed/?term=Melby%20KK%5BAuthor%5D&cauthor=true&cauthor_uid=23083455) Resistance rates of metronidazole and other antibacterials in Helicobacter pylori from previously untreated patients in Norway. [APMIS.](https://www.ncbi.nlm.nih.gov/pubmed/?term=Larsen%2BAL%2Band%2BRagnhildstveit) 2013 Apr;121(4):353-8
10 [Malfertheiner P,](https://www.ncbi.nlm.nih.gov/pubmed/?term=Malfertheiner%20P%5BAuthor%5D&cauthor=true&cauthor_uid=27707777) [Megraud F,](https://www.ncbi.nlm.nih.gov/pubmed/?term=Megraud%20F%5BAuthor%5D&cauthor=true&cauthor_uid=27707777) [O'Morain CA.](https://www.ncbi.nlm.nih.gov/pubmed/?term=O%27Morain%20CA%5BAuthor%5D&cauthor=true&cauthor_uid=27707777) Management of Helicobacter pylori infection-the Maastricht V/Florence Consensus Report [Gut.](https://www.ncbi.nlm.nih.gov/pubmed/27707777) 2017 Jan;66(1):6-30. doi: 10.1136/gutjnl- 2016-312288. Epub 2016 Oct 5.
11 [Katelaris P,](https://www.ncbi.nlm.nih.gov/pubmed/?term=Katelaris%20P%5BAuthor%5D&cauthor=true&cauthor_uid=28345276) [Katelaris A](https://www.ncbi.nlm.nih.gov/pubmed/?term=Katelaris%20A%5BAuthor%5D&cauthor=true&cauthor_uid=28345276). A prospective evaluation of levofloxacin based triple therapy for refractory Helicobacter pylori infection in Australia. [Intern Med J.](https://www.ncbi.nlm.nih.gov/pubmed/28345276) 2017 Mar 27. doi: 10.1111/imj.13432.
12 Nestegard O, Johnsen KM, Sørbye S, Halvorsen FA, Tønnessen T, Paulssen E, Melby K, Goll R, Florholmen J. Clinical characterization of *Helicobacter pylori* infected patients 15 years after unsuccessful eradication. Research Article | published 23 Sep 2020 PLOS ONE <https://doi.org/10.1371/journal.pone.0238944>
13 Francis Mégraud, Chloé Alix, Paul Charron, Lucie Bénéjat, Astrid Ducournau, Emilie Bessède, Philippe Lehours. Survey of the antimicrobial resistance of Helicobacter pylori in France in 2018 and evolution during the previous 5 years. Helicobacter. 2021;26: e12767
14 Alba C, Blanco A, Alarcon T. Antibiotic resistance in Helicobacter pylori Curr Opin Infect Dis 2017,30:Epub 2017
15 [Bich N Dang](https://pubmed.ncbi.nlm.nih.gov/?term=Dang%2BBN&cauthor_id=28465548) , [David Y Graham](https://pubmed.ncbi.nlm.nih.gov/?term=Graham%2BDY&cauthor_id=28465548)

Helicobacter pylori infection and antibiotic resistance: a WHO high priority? Nat Rev Gastroenterol Hepatol . 2017 Jul;14(7):383-384.

16 A[ntibiotic usage ESDC report 2015 A](https://scholar.google.no/scholar?q=antibiotic%2Busage%2B%2BECDC%2Breport%2B2015&hl=no&as_sdt=0&as_vis=1&oi=scholart)nnual reports and protocols on antimicrobial consumption (europa.eu)
17 Malfertheiner P, Megraud F, O’Morain C, et al. Current European concepts in the management of Helicobacter pylori infection: the Maastricht Consensus Report. The European Helicobacter Pylori Study Group (EHPSG). Eur J Gastroenterol Hepatol 1997;9:1e2.
18 Megraud F, Coenen S, Versporten A, Kist M, Lopez-Brea M, Hirschl AM, Andersen LP, Goossens H, Glupczynski Y; Study Group participants.	Gut. 2013 Jan;62(1):34-42.

Helicobacter pylori resistance to antibiotics in Europe and its relationship to antibiotic consumption.

19 Steinbakk M, Moyeri B, Melby KK. Antibiotic sensitivity pattern in Helicobacter pylori from previously untreated patients in South-Eastern Norway 2018. (Poster) Nordic Soc Antimicrob Resistance Conference, Trondheim, Norway 2019.
20 The National Prescription-Based Medicines Register (Prescription Register) Prescription Norwegian [Database/reseptregister.data@fhi.no](mailto:Database/reseptregister.data@fhi.no)
21 Nichols AG, et al on the behalf of the Hp-EuReg Investigators. Pan-European Registry on H.pylori Management (Hp-Eu-Reg): Bacterial resistance of 2241 H.pylori isolates.

Helicobacter 2017:22,Suppl 1, 56.

22 [Asfeldt AM](https://www.ncbi.nlm.nih.gov/pubmed/?term=Asfeldt%20AM%5BAuthor%5D&cauthor=true&cauthor_uid=15545164), [Løchen ML,](https://www.ncbi.nlm.nih.gov/pubmed/?term=L%C3%B8chen%20ML%5BAuthor%5D&cauthor=true&cauthor_uid=15545164) [Straume B,](https://www.ncbi.nlm.nih.gov/pubmed/?term=Straume%20B%5BAuthor%5D&cauthor=true&cauthor_uid=15545164) [Steigen SE](https://www.ncbi.nlm.nih.gov/pubmed/?term=Steigen%20SE%5BAuthor%5D&cauthor=true&cauthor_uid=15545164), [Florholmen J](https://www.ncbi.nlm.nih.gov/pubmed/?term=Florholmen%20J%5BAuthor%5D&cauthor=true&cauthor_uid=15545164), [Goll R](https://www.ncbi.nlm.nih.gov/pubmed/?term=Goll%20R%5BAuthor%5D&cauthor=true&cauthor_uid=15545164), [Nestegard O](https://www.ncbi.nlm.nih.gov/pubmed/?term=Nestegard%20O%5BAuthor%5D&cauthor=true&cauthor_uid=15545164), [Paulssen EJ](https://www.ncbi.nlm.nih.gov/pubmed/?term=Paulssen%20EJ%5BAuthor%5D&cauthor=true&cauthor_uid=15545164). Accuracy of a monoclonal antibody-based stool antigen test in the diagnosis of Helicobacter pylori infection. [Scand J Gastroenterol.](https://www.ncbi.nlm.nih.gov/pubmed/15545164) 2004 Nov;39(11):1073-7.
23 Gunnarsdottir AI, Gudjonsson H, Hardardottir H, Jonsdottir KD, Bjornsson ES. Antibiotic susceptibility of Helicobacter pylori in Iceland. Infect Dis (Lond). 2017 Sep;49(9):647-654. doi: 10.1080/23744235.2017.1317359. Epub 2017 Apr 25.
24 Najmeh Salehi, Bahareh Attaran, Negin Eskini, Maryam Esmaeili, Atefeh Sharifirad, Mehdi Sadeghi, Marjan Mohammadi. New insights into resistance of Helicobacter pylori against third and fourth‐ generation fluoroquinolones: A molecular docking study of prevalent GyrA mutations. Helicobacter. 2019;24:e12628.
25 Alessia Savoldi, Elena Carrara, David Y. Graham, Michela Conti, Evelina Tacconelli. Prevalence of Antibiotic Resistance in Helicobacter pylori: A Systematic Review and Meta-analysis in World Health Organization Regions.

Gastroenterology. 2018 November; 155(5): 1372–1382.e17. i:10.1053/j.gastro.2018.07.007.

26 Sinead M., Colm O’Morain, and Deirdre McNamara. Helicobacter pylori resistance to current therapies. Curr Opin Gastroenterol 2019, 35:6-13
27 Sheila E. Crowe, Helicobacter pylori Infection.N Engl J Med 2019;380:1158-65.DOI: 10.1056/NEJMcp1710945

**Table 1.** Examinations for *H. pylori* by conventional culture of gastric biopsies.

| **Study group** | Group 1 | | Group 2 | |
| --- | --- | --- | --- | --- |
|  | Before  treatment | After  treatment | Before  treatment | After treatment |
| Cultures | 42 | 37 | 50 | 42 |
| negative | 16 (38.1%) | 35 (94.5%) | 8 (16.0%) | 33 (78.6%) |
| positive | 26 (61.9%) | 2 (4.5%) | 42 (84.0%) | 9 (21.4%) |

Results from *H. pylori* cultures of gastric mucosal biopsies before or after treatment in the two study groups. Group 1: resistant *H. pylori*, Group 2: naïve *H. pylori*.

Numbers are n or n (%). For further details, see text.

**Table 2.** Antimicrobial resistance in *H. pylori* cultures from …. ~~the two study groups~~.

| **Group 1** | Before treatment (n = 26) | | | After treatment (n= 2) | | |
| --- | --- | --- | --- | --- | --- | --- |
|  | Sensitive | Resistant | Intermediate | Sensitive | Resistant | Intermediate |
| Amoxicillin | 26 | 0 | 0 | 2 | 0 | 0 |
| Clarithromycin | 16 | 10 (38.5%) | 0 | 2 | 0 | 0 |
| Metronidazole | 7 | 16 (69.5%) | 0 | 1 | 1 (50%) | 0 |
| Tetracycline | 23 | 2 (8.0%) | 0 | 2 | 0 | 0 |

Table 3. Antimicrobial resistance in *H. pylori* cultures from…

| **Group 2** | Before treatment (n = 42) | | | After treatment (n = 9; valid = 7) | | |
| --- | --- | --- | --- | --- | --- | --- |
|  | Sensitive | Resistant | Intermediate | Sensitive | Resistant | Intermediate |
| Amoxicillin | 42 | 0 | 0 | 7 | 0 | 0 |
| Clarithromycin | 38 | 4 (10.5%) | 0 | 5 | 1 (14.1%) | 1 |
| Metronidazole | 25 | 16 (38.1%) | 1 | 4 | 3 (42.8%) | 0 |
| Tetracycline | 36 | 5 (11.9%) | 1 | 7 | 0 | 0 |

Tests for antimicrobial resistance in cultures of *H. pylori* in the to study groups, before and after treatment. Group 1: resistant *H. pylori*, Group 2: naïve *H. pylori*. Two of the cultures in Group 2 “After treatment” did not yield valid test results. Numbers are n or n (%).

Figure	[Click here to access/download;Figure;CONSORT 2010 Flow Diagram (1) 110821.doc](https://www.editorialmanager.com/pone/download.aspx?id=29629320&guid=c6b57ac9-3ce3-4c1e-9953-839c2f6011bb&scheme=1)

# CONSORT 2010 Flow Diagram

**Enrollment**

**Allocation**

Allocated to intervention (n= 42 )

- Received allocated intervention (n=42 )
- Did not receive allocated intervention (give reasons) (n= 0 )

Allocated to intervention (n= 50 )

- Received allocated intervention (n= 50 )
- Did not receive allocated intervention (give reasons) (n= 0 )

**Follow-Up**

Lost to follow-up (give reasons) (n=0 )

Lost to follow-up (give reasons) (n= 0)

Discontinued intervention (give reasons) (n= 0

)

Discontinued intervention (give reasons) (n= 0

)

**Analysis**

Analysed (n= 42 )

- Excluded from analysis (give reasons) (n= 0

)

Analysed (n= 50 )

- Excluded from analysis (give reasons) (n= 0

)

Randomized (n= 92 )

Excluded (n= 2 )

- Not meeting inclusion criteria (n= 0 )
- Declined to participate (n= 2 )
- Other reasons (n= 0)

Assessed for eligibility (n= 94 )

Figure	[Click here to](https://www.editorialmanager.com/pone/download.aspx?id=29629321&guid=d73f5096-6632-4d35-b87b-b981acf9bc70&scheme=1)

[access/download;Figure;trendstatement_TREND_Checklist.pdf](https://www.editorialmanager.com/pone/download.aspx?id=29629321&guid=d73f5096-6632-4d35-b87b-b981acf9bc70&scheme=1)

### TREND Statement Checklist

| **Paper**  **Section/ Topic** | **Item No** | **Descriptor** | **Reported?** | |
| --- | --- | --- | --- | --- |
|  |  |  |  | **Pg #** |
| **Title and Abstract** | | | | |
| Title and Abstract | 1 | Information on how unit were allocated to interventions |  | 2 |
|  |  | Structured abstract recommended |  | 2-3 |
|  |  | Information on target population or study sample |  | 2-3 |
| **Introduction** | | | | |
| Background | 2 | Scientific background and explanation of rationale |  | 4 |
|  |  | Theories used in designing behavioral interventions |  | 4 |
| **Methods** | | | | |
| Participants | 3 | Eligibility criteria for participants, including criteria at different levels in recruitment/sampling plan (e.g., cities, clinics, subjects) |  | 5-6 |
|  |  | Method of recruitment (e.g., referral, self-selection), including the sampling method if a systematic sampling plan was implemented |  | 5-6 |
|  |  | Recruitment setting |  | 5-6 |
|  |  | Settings and locations where the data were collected |  | 5-6 |
| Interventions | 4 | Details of the interventions intended for each study condition and how and when they were actually administered, specifically including: | N.A. |  |
|  |  | o Content: what was given? |  |  |
|  |  | o Delivery method: how was the content given? |  |  |
|  |  | o Unit of delivery: how were the subjects grouped during delivery? |  |  |
|  |  | o Deliverer: who delivered the intervention? |  |  |
|  |  | o Setting: where was the intervention delivered? |  |  |
|  |  | o Exposure quantity and duration: how many sessions or episodes or events were intended to be delivered? How long were they intended to last? |  |  |
|  |  | o Time span: how long was it intended to take to deliver the intervention to each unit? |  |  |
|  |  | o Activities to increase compliance or adherence (e.g., incentives) |  |  |
| Objectives | 5 | Specific objectives and hypotheses | N.A. |  |
| Outcomes | 6 | Clearly defined primary and secondary outcome measures |  | 6 |
|  |  | Methods used to collect data and any methods used to enhance the quality of measurements |  | 6-7 |
|  |  | Information on validated instruments such as psychometric and biometric properties |  |  |
| Sample Size | 7 | How sample size was determined and, when applicable, explanation of any interim analyses and stopping rules |  | 5-6 |
| Assignment Method | 8 | Unit of assignment (the unit being assigned to study condition, e.g., individual, group, community) |  | 5-6 |

**TREND Statement Checklist**

|  |  | Method used to assign units to study conditions, including details of any restriction (e.g., blocking, stratification, minimization) |  |  |
| --- | --- | --- | --- | --- |
|  |  | Inclusion of aspects employed to help minimize potential bias induced due to non-randomization (e.g., matching) |  |  |

| Blinding (masking) | 9 | Whether or not participants, those administering the interventions, and those assessing the outcomes were blinded to study condition assignment; if so, statement regarding how the blinding was accomplished and how it was assessed. | N.A. |  |
| --- | --- | --- | --- | --- |
| Unit of Analysis | 10 | Description of the smallest unit that is being analyzed to assess intervention effects (e.g., individual, group, or community) |  | 6-7 |
|  |  | If the unit of analysis differs from the unit of assignment, the analytical method used to account for this (e.g., adjusting the standard error  estimates by the design effect or using multilevel analysis) |  |  |
| Statistical Methods | 11 | Statistical methods used to compare study groups for primary methods outcome(s), including complex methods of correlated data | N.A. |  |
|  |  | Statistical methods used for additional analyses, such as a subgroup analyses and adjusted analysis |  |  |
|  |  | Methods for imputing missing data, if used |  |  |
|  |  | Statistical software or programs used |  |  |
| **Results** | | | | |
| Participant flow | 12 | Flow of participants through each stage of the study: enrollment, assignment, allocation, and intervention exposure, follow-up, analysis (a  diagram is strongly recommended) |  | 6-7 |
|  |  | o Enrollment: the numbers of participants screened for eligibility, found to be eligible or not eligible, declined to be enrolled, and enrolled in the study |  |  |
|  |  | o Assignment: the numbers of participants assigned to a study condition |  |  |
|  |  | o Allocation and intervention exposure: the number of participants assigned to each study condition and the number of participants who received each intervention |  |  |
|  |  | o Follow-up: the number of participants who completed the followup or did not complete the follow-up (i.e., lost to follow-up), by study  condition |  |  |
|  |  | o Analysis: the number of participants included in or excluded from the main analysis, by study condition |  |  |
|  |  | Description of protocol deviations from study as planned, along with reasons |  |  |
| Recruitment | 13 | Dates defining the periods of recruitment and follow-up |  | 6-7 |
| Baseline Data | 14 | Baseline demographic and clinical characteristics of participants in each study condition |  | 5-6 |

### TREND Statement Checklist

|  |  | Baseline characteristics for each study condition relevant to specific disease prevention research |  | 5-6 |
| --- | --- | --- | --- | --- |
|  |  | Baseline comparisons of those lost to follow-up and those retained, overall and by study condition |  | 5-6 |
|  |  | Comparison between study population at baseline and target population of interest |  | 5-6 |
| Baseline equivalence | 15 | Data on study group equivalence at baseline and statistical methods used to control for baseline differences | N.A. |  |
| Numbers analyzed | 16 | Number of participants (denominator) included in each analysis for each study condition, particularly when the denominators change for different  outcomes; statement of the results in absolute numbers when feasible |  | 5-6 |
|  |  | Indication of whether the analysis strategy was “intention to treat” or, if not, description of how non-compliers were treated in the analyses |  | 5-6 |
| Outcomes and estimation | 17 | For each primary and secondary outcome, a summary of results for each estimation study condition, and the estimated effect size and a confidence  interval to indicate the precision |  | 6-7 |
|  |  | Inclusion of null and negative findings |  | 6-7 |
|  |  | Inclusion of results from testing pre-specified causal pathways through which the intervention was intended to operate, if any |  | 6-7 |
| Ancillary analyses | 18 | Summary of other analyses performed, including subgroup or restricted analyses, indicating which are pre-specified or exploratory |  | 6-7 |
| Adverse events | 19 | Summary of all important adverse events or unintended effects in each study condition (including summary measures, effect size estimates, and confidence intervals) |  | 6-7 |
| **DISCUSSION** | | | | |
| Interpretation | 20 | Interpretation of the results, taking into account study hypotheses, sources of potential bias, imprecision of measures, multiplicative analyses, and  other limitations or weaknesses of the study |  | 7-10 |
|  |  | Discussion of results taking into account the mechanism by which the intervention was intended to work (causal pathways) or alternative  mechanisms or explanations |  | 7-10 |
|  |  | Discussion of the success of and barriers to implementing the intervention, fidelity of implementation |  | 7-10 |
|  |  | Discussion of research, programmatic, or policy implications |  | 7-10 |
| Generalizability | 21 | Generalizability (external validity) of the trial findings, taking into account the study population, the characteristics of the intervention, length of follow-up, incentives, compliance rates, specific sites/settings involved in  the study, and other contextual issues |  | 7-10 |
| Overall Evidence | 22 | General interpretation of the results in the context of current evidence and current theory |  | 7-10 |

*From:* Des Jarlais, D. C., Lyles, C., Crepaz, N., & the Trend Group (2004). Improving the reporting quality of

### TREND Statement Checklist

nonrandomized evaluations of behavioral and public health interventions: The TREND statement. *American Journal of Public Health*, 94, 361-366. For more information, visit: <http://www.cdc.gov/trendstatement/>
