# Supplementary material for: Helicobacter pylori resistance to antibiotics before and after treatment: Incidence of eradication failure
Source: PLoS One. 2022 Apr 20;17(4):e0265322. doi: 10.1371/journal.pone.0265322 (PMC9020706; doi:10.1371/journal.pone.0265322)
Supplement: Attachment — Submitted filename: Response to reviewers 090122.docx [file pone.0265322.s006.docx]

Dear editor and reviewers

We have done changes in the article due to proposals from editor end reviewers.

That is the title of the article, abstract and keywords.

In introduction we have taken in relationship between *H. pylori* and gastric cancer. There is a new reference for this.

WHOs concerning about *H. pylori* and antibiotic resistance is mentioned.

In material and methods we have done changes as indicated from the reviewers and there is information about statistic there.

In results we have specified the inclusion and exclusion criteria.

In discussion we have added percentage of resistens for tetracycline.

In tables we have changed text and have put in sensitivity in percentage for each antibiotics.

MICs are in a Supporting Information file.

Hønefoss 12. Jan. 2022

On behalf of the authors

With kind regards

Oddmund Nestegard
